# Application of non-destructive testing methods for assessing fracture resistance in dental ceramics: the sound harvesting test

**DOI:** 10.3389/fdmed.2024.1409150

**Published:** 2024-06-06

**Authors:** Camille Haddad, Jean Gebran, Amine El Zoghbi

**Affiliations:** ^1^ Department of Prosthodontics and Occlusion, Saint Joseph University, Beirut, Lebanon; ^2^ Charge d'enseignement, IESAV, Saint Joseph University, Beirut, Lebanon; ^3^ Department of Prosthodontics and Occlusion, Laboratoire de Recherche Cranio-Faciale-Unite d'anomalie Cranio-Faciale, Saint Joseph University, Beirut, Lebanon

**Keywords:** monolithic zirconia, fracture resistance test, acoustic emission testing, sound harvesting test, brittle materials

## Abstract

**Introduction:**

Evaluating the fracture resistance of dental ceramics such as monolithic zirconia crowns is crucial for assessing their durability. Conventional destructive laboratory tests often fail to accurately evaluate the timing and failing crack formation of these brittle materials. Non-destructive testing methods, such as acoustic emission testing (AET), offers an alternative by providing valuable data on material properties without causing damage to the samples.The *in vitro* study aimed to evaluate the sensitivity of a sound harvesting modified acoustic emission testing by comparing the fracture resistance of posterior monolithic zirconia crowns (MZCs) measured via the modified set up with that of a conventional fracture toughness test.

**Material and methods:**

A modified acoustic emission set up, the sound harvesting test (SHT), featuring a condenser microphone, an amplifier, a custom audio chipset and a cut-off switch integrated into a universal testing machine, was compared to a conventional fracture toughness test to measure fracture loads on 50 posterior monolithic zirconia crowns divided in two groups.

**Results:**

The sound harvesting test recorded a mean fracture load of 1,108.99 N, significantly less than the 1,292.52 N measured with the conventional test, indicating a more sensitive detection of fractures. Statistically significant differences (*p* < 0.05) were observed between the two groups.

**Conclusion:**

Despite its limitations, the study suggests considering sound harvesting testing as an potential alternative for fracture load testing of dental brittle materials due to its ability to identify failures at lower loads enhancing therefore a more accurate evaluation of the behavior of dental materials. However, further testing on a broader range of dental materials is warranted to improve result accuracy and applicability.

## 1 Introduction

Over the past decades, ceramic materials have gained significant importance in dentistry due to their optical properties and biocompatibility, making them ideal for dental prostheses notably, monolithic zirconia material commonly used in the posterior crowns due to its high strength compared to all ceramic materials which contribute to the stability and longevity of posterior fixed partial dentures (FPDs) (1).

However, ceramics are naturally brittle, with limited tensile strength and susceptibility to time-dependent stress failure when subjected to various forces within the oral cavity, including masticatory forces, occlusal stresses, and thermal changes (2). As a result, fracture resistance is a critical aspect of their performance, as it directly impacts the longevity and durability of dental restoration (3, 4).

In vitro studies are crucial for evaluating the potential clinical performance of dental materials before embarking on costly and time-consuming clinical investigations (5). Assessing their fracture resistance allows clinicians and researchers to evaluate their suitability for different clinical applications and to predict their clinical performance over time. This evaluation process involves testing the materials under simulated loading conditions to measure their resistance to crack propagation and failure (6).

Several destructive testing (DT) techniques, including strength tests and fracture toughness tests (FT), are used to evaluate the performance and properties of dental ceramics. Using a universal testing machine (UTM) with a stainless-steel indenter, the FT test is based on applying a compressive load to the occlusal surface of samples, to measure the force required to cause fracture. However, the DT, while effective, have several limitations. First, these tests involve sample destruction, rendering the samples unusable for further testing, which can be costly for materials that are expensive or available in limited quantities. Second, DT provides only a single-point evaluation of the material's properties at a specific moment, failing to capture early changes, such as crack initiation, over time due to factors like aging in clinical situations (7). Finally, DT also offers only a limited assessment of failure modes, focusing primarily on determining the maximum failure strength without providing detailed insights into specific failure mechanisms. To address these limitations, non-destructive testing (NDT) methods offer an alternative approach to evaluating brittle dental ceramics, preserving their functionality while assessing structural integrity, quality, and performance (8).

NDT techniques are valuable tools for assessing the integrity of structures and materials in various industries, including dentistry and ceramic production. These methods enable researchers to evaluate materials or components without causing damage, offering significant cost savings, and ensuring the quality of engineered systems and products. In ceramic production, NDT methods are crucial for detecting structural defects without causing damage, although certain key areas still lack viable testing methods, emphasizing the need for the development of additional accurate and effective NDT techniques to ensure ceramic product quality (9, 10). Commonly used NDT methods include visual inspection, penetration testing, ultrasonic testing, radiographic testing, infrared thermography, laser ultrasonics, x-rays, optical coherence tomography, laser ultrasonics, computed tomography, and acoustic emission testing (AET) (11).

The fundamental principle of the AET involves harvesting electrical energy from mechanical vibrations generated by sound waves (10) effectively identifying the initiation and progression of failure in brittle materials while maintaining the integrity of the tested samples. It is used to assess properties such as fracture strength. Integrated with conventional static fracture test machines, AET can determine the onset of failure, locate the initial damage site, track damage propagation, and expose the complex mechanisms leading to material failure (7–9).

One variant of AET involves using a microphone instead of an ultrasound sensor to harvest the early sound emitted by the crack formation in failing ceramic material. When converted to electrical signals, the emitted noises can be used to transform traditional destructive strength testing into NDT by activating a cutoff switch to halt the UTM load on crack initiation (12).

The fundamental of sound harvesting test (SHT), is the transmission of an electrical signal from a brittle material such as zirconia to a microphone, amplifier, and switch breaker; this process involves various factors influencing the speed of transmission. These factors include the distance between the material and the microphone, microphone sensitivity, amplifier quality, and switch breaker response time (13). Assuming that high-quality elements with low latency are used, the signal's travel time can be estimated. Sound waves travel quickly through materials such as ceramic, but once reaching a microphone, they are converted into electrical signals transmitted through a cable to an amplifier. The amplifier processes the signal and sends it to the switch breaker, ideally with minimal latency.

The speed of the signal in the cable is influenced by the cable length, quality, and electrical properties such as resistance and capacitance. Additionally, the amplifier's processing time depends on its design and settings, with modern amplifiers typically having low latency (14). Because they travel at the speed of light, electric signal transmission through wires is much faster than the speed of sound in air. Material characteristics, such as composition and stress conditions, affect crack propagation speed. The electrical circuit's ability to rapidly stop the test upon crack initiation is vital for accurate data collection (15). By integrating a high-sensitivity microphone within the fracture toughness test, we can precisely monitor the noise emissions generated by the samples during loading. This setup, coupled with a custom-designed “cut-off” switch system, enables automatic halting of the load process upon detecting abnormal sounds indicative of crack initiation.

The objective of this *in vitro* study was to evaluate the sensitivity of a modified acoustic emission testing, the sound harvesting test (SHT), by comparing the fracture resistance of posterior monolithic zirconia crowns (MZCs) measured via the technique with that of a conventional fracture toughness test.

## 2 Materials and methods

### 2.1 Sample preparation

A total of 50 zirconia monolithic crowns (Initial Zirconia Disk® monolithic translucent produced by GC®, Leuven, Belgium) were tested in this experiment. They were evenly distributed into two groups: 25 crowns underwent SHT, while the remaining 25 served as a control group with a FT. The study employed a blinded assessment for unbiased evaluation.

An operator milled a single intact artificial mandibular first molar (Frasaco®, Tettnang, Germany) with a turbine handpiece and diamond burs of different diameters. The occlusal surface area was reduced by 1.5 mm (functional cusps). The axial walls were tapered by four degrees and reduced by 1.2 mm (Komet® 8862, Lemgo, Germany). The artificial tooth was prepared with a feather-edge margin of 0.5 mm (13). All edges were rounded and polished with a handpiece micromotor, silicone polishing burs of different grain diameters, and a polishing brush with polishing paste (Dialux® blanc, Salisbury, UK). The tooth that had been prepared was subsequently placed horizontally along the customized metallic mold's axis, which held cold-cure acrylic resin with dimensions measuring 2.5 × 2.5 × 3 mm^3^.

The die, including an artificially prepared tooth and acrylic base, was scanned by a laboratory scanner (Dental Wings, Exocad, 3 Shape®, Montreal, CA, USA). The standard tessellation language (STL) file was analyzed using computer-aided design (CAD) software (Mayka Dental V6, Picasoft®, Yangon, Burma).

The 3D virtual die was adjusted in the CAD software according to the manufacturer's directives with a 0.5 mm feather-edge margin. By using the gap thickness tool in the CAD software, a space of 40 microns dedicated to the cement was formed. Based on the CAD die, 50 polymethylmethacrylate (PMMA) dies were printed (Formlabs 2®, Somerville, MA, USA) by a 3D printing machine.

The MZCs were milled by a five-axis milling unit (Kavo Everest®, Charlotte, NC, USA) from four monolithic zirconia discs measuring 98 mm × 16 mm (Initial Zirconia Disk® monolithic translucent by GC®). The MZCs were sintered at 1,500°C in a sintering furnace according to the manufacturer's instructions.

After sandblasting (50-micron 1.5 bar), MZC were then cemented to printed PMMA models with a universal dual cure resin cement (G Cem one®, GC®) light cured for 3 s with a curing light machine offering a light intensity of 2,500 mW/cm^2^ (Woodpecker iLED®).

A 1 mm rubber cylinder was made and placed between the indenter tool and the crown to prevent direct damage and evenly distribute the forces. To secure the sample on the die, a universal testing machine applied a 20 Newton vertical load on the top surface. The same operator repeated this process for each MZC- PMMA die cementation. All samples were kept in an incubator for 7 days prior to mechanical testing (15).

A thermocycling procedure was then carried out. It consisted of 500,000 cycles alternating between 5 and 55°C. The immersion time in each bath was 20 seconds, and the transfer time was 5 seconds (14, 15).

### 2.2 Fracture toughness tests

To prevent Hertzian damage during both tests, a 2 mm urethane rubber cylinder was placed between the indenter and the sample.

To perform the conventional fracture toughness test with control group, a ball-shaped indenter was used to create an axial load on the occlusal surface of the samples (3). The compressive load was applied at a crosshead speed of 0.5 mm per minute until fracture. Load values were recorded by the UTM (Laboratoire de Recherche Cranio-faciale-Saint Joseph university) (16).

**Figure 1:**
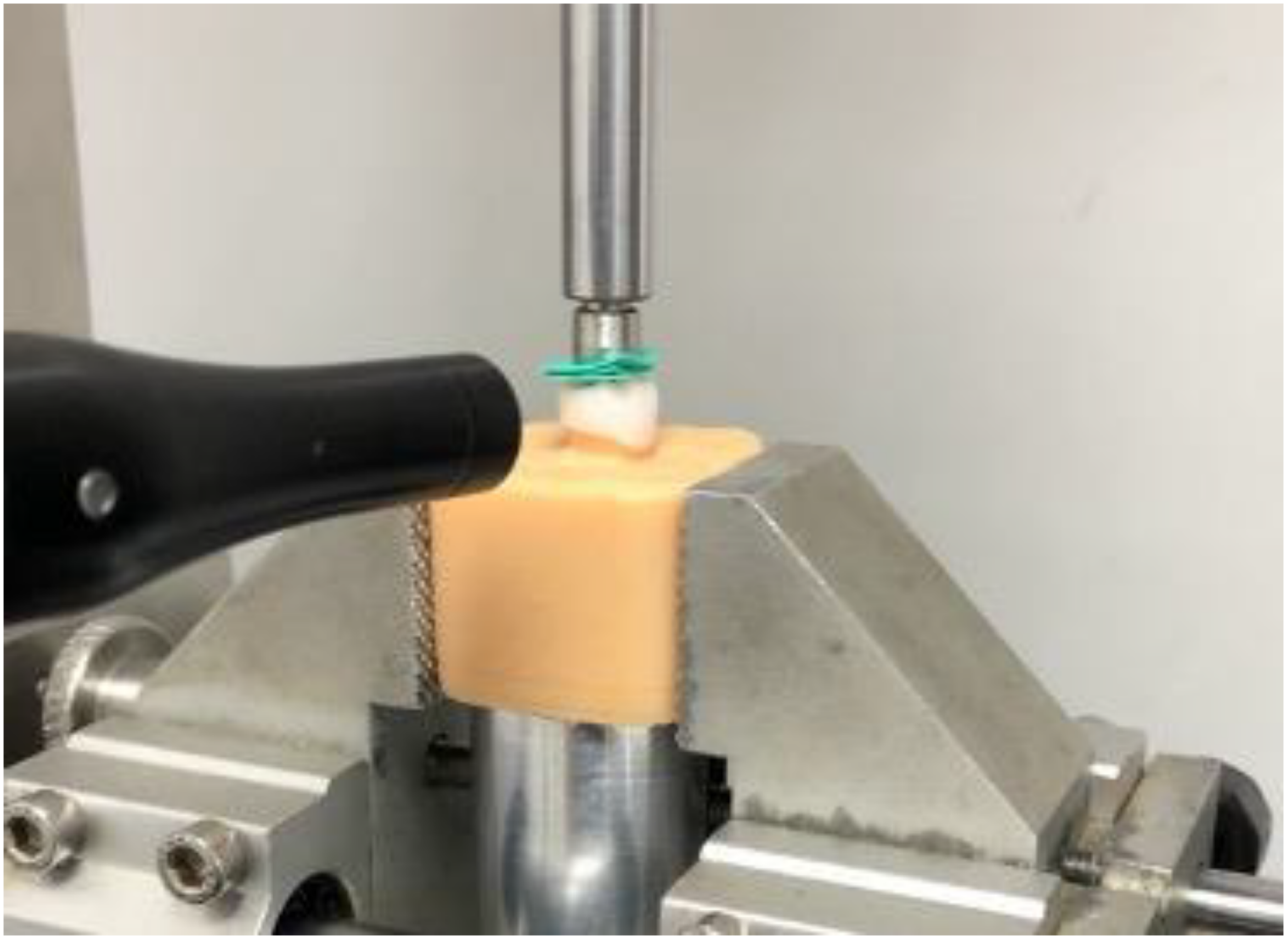
Condenser microphone, urethan sheet, PMMA base and a MZC sample in the SHT setup.

Sound Harvesting test was carried out by placing a microphone near the sample within the UTM (16) (YLE® GmbH, Waldstraße Bad König, Germany) (17). Specifically, a condenser microphone (MiniSPL®, NTI) was positioned 1 cm away from the sample (18). It was connected to an amplifier (Avalon Design 737*®*, Nashville, TN, USA) in wich was integrated a motherboard chipset (Figure 1) (19).

**Figure 2:**
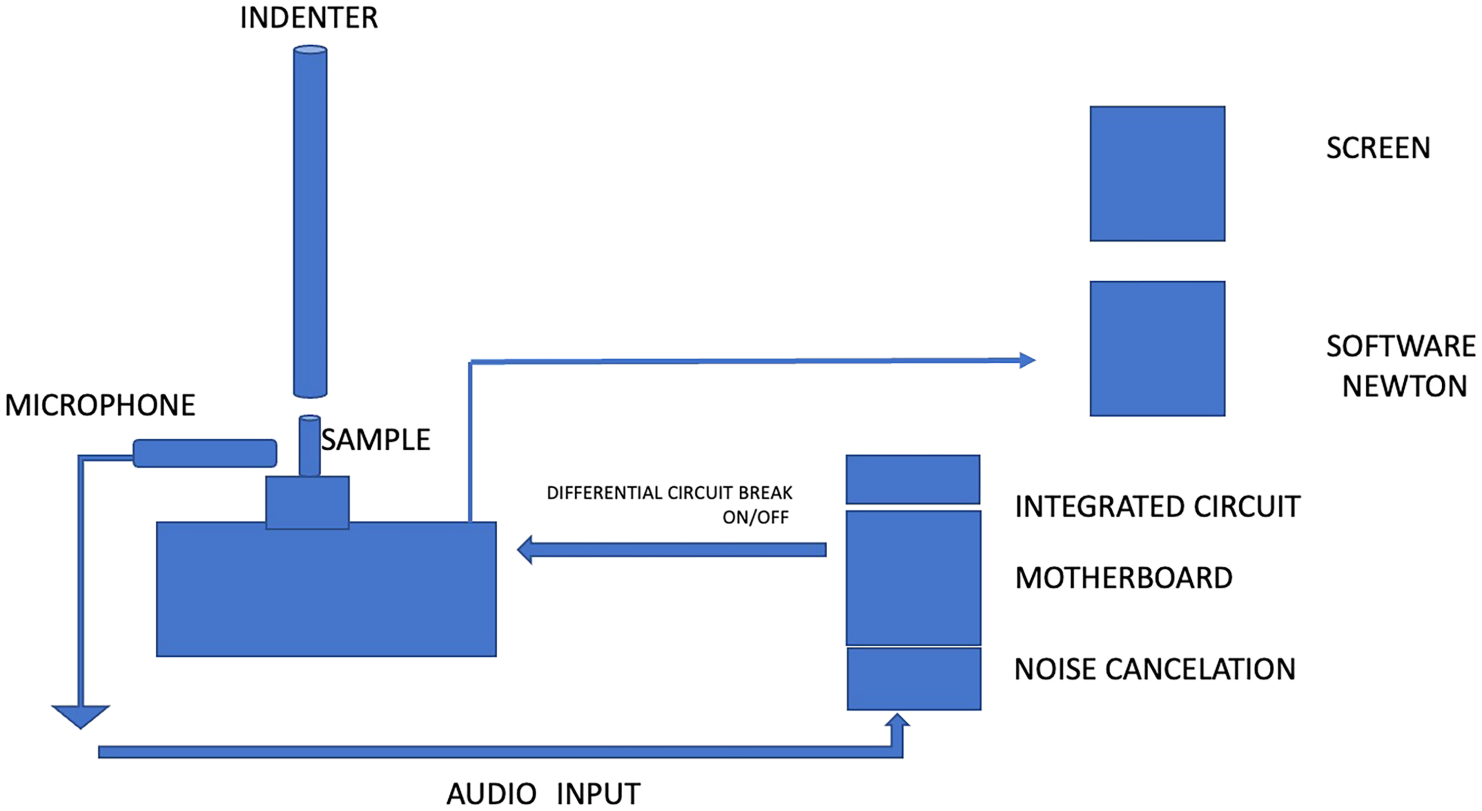
The sound harvesting test setup.

To safeguard the samples from destruction, a custom made “cut-off” switch system was integrated within the UTM, stopping the test upon detecting the specific sound of a crack forming (Figure 2).

To ensure the UTM's operational sounds did not interfere, a preload of 20 N was applied to secure the crown onto its PMMA base (9). Subsequently, the recording was reinitialized, and the desired test commenced.

Throughout the test, the amplifier continuously monitored for any deviations from normal machine noise. The chipset was programmed to differentiate between normal UTM noise descending and crack sounds emitted by the sample. Upon detecting a crack sound, the chipset triggered an electric command through the cutoff switch to halt the UTM, automatically recording the load values in Newton. To minimize external sound interference, corrugated foam sheets (Cactus® USA) were used for noise cancellation during the tests (20).

The SHT data were collected by the UTM software for analysis and storage.

### 2.3 Visual inspection of cracks

**Figure 3:**
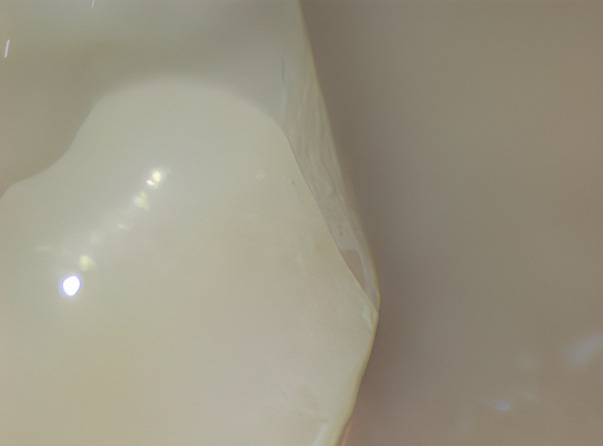
Detected early crack on MZC.

Following the static load test, cracks in the samples were identified, and subsequent meticulous examination under a low-magnification microscope (Leica Microsystems®, Wetzlar, Germany) was conducted. Photographic documentation of the samples was carried out using a DSL camera (Nikon®, Tokyo, Japan) for further analysis of the crack location or potential fractures. Photographs of all the samples were taken in various positions and analyzed to determine the location of the crack or eventual fracture (Figure 3).

### 2.4 Statistical analysis

The statistical analyses for the study were performed with SPSS Statistics for Windows, version 25.0 (IBM® Corp, Armonk, NY, USA). Independent samples *t*-testing was conducted to examine whether there was a significant difference in the load means between the two tested groups. In every situation, a *p*-value less than 0.05 was considered statistically significant.

## 3 Results

Table 1 presents fracture loads (in Newtons, N) for MZCs obtained using SHT and the conventional technique. With SHT, the fracture loads ranged from 217.99 N to 1,748.00 N, with a mean of 1,108.99 N and standard deviation (SD) of 327.89. Without SHT, the fracture loads ranged from 840.00 N to 1,840.00 N, with a mean of 1,292.52 N and SD of 271.42. Statistical analysis revealed a significant difference (*p* = 0.036) between the two techniques, as determined by the independent samples *t*-test.

**Table 1:** Fracture loads in newtons (N) obtained for the 2 testing methods (*n* = 50).

	SHT				Conventional				*p*-Value
	Min	Max	Mean	SD	Min	Max	Mean	SD	
MZC	217.99	1748.00	1108.99	327.89	840.00	1840.00	1292.52	271.42	**0**.**036**

A *p*-value less than 0.05 was considered statistically significant.

### 3.1 Classification of crack/fracture

Table 2 provides a comparison between the SHT method and the conventional method for detecting fractures and cracks.

**Table 2:** Crack to fracture ratio with the 2 testing methods.

	SHT	Conventional	Total
	*n* (%)	*n* (%)	*n*
Fracture	6 (24%)	18 (72%)	24 (48%)
Crack	19 (76%)	7 (28%)	26 (52%)
Total	25 (50%)	25 (50%)	50 (100%)

Percentage of cracks (*n* = 50).

Sound harvesting test detected 6 out of 25 fractures (24%), while the conventional method detected 18 out of 25 fractures (72%), resulting in a total of 24 fractures out of 50 samples (48%).

For cracks, SHT detected 19 out of 25 cases (76%), whereas with the conventional method only 2 samples out of 25 cracked (28%).

## 4 Discussion

In the presented study, the effectiveness of a sound harvesting test was examined for its ability to detect cracks in brittle dental ceramics such as monolithic zirconia crowns. The performance of SHT in monitoring fracture events was evaluated and compared with conventional fracture toughness test that measure fracture loads in Newtons (N).

The results revealed that SHT identified lower mean fracture loads of 1,108.99 N compared to 1,292.52 N with standard tests, showcasing SHT's higher sensitivity in detecting fractures at earlier stages. Statistical analysis showed that these differences were significant, supporting the hypothesis that SHT can provide more accurate fracture load assessments.

Statistical analyses supported by significant *p*-values emphasize the non-random nature of these differences, aligning with prior research, such as Jin and al (21). which emphasized the influence of detection methods on crown fracture load.

For cracks, SHT detected (76%), whereas with the conventional method only 2 samples out of 25 cracked (28%). Overall, SHT exhibited higher sensitivity in detecting cracks.

Regarding the selection of research materials, careful consideration was granted to ensure optimal sound isolation, transmission, and collection. The die material was chosen to replicate the characteristics of natural teeth. PMMA resin was chosen due to its similarity to natural teeth in terms of the modulus of elasticity and acoustic response (22).

A study by Nakamura et al. revealed that the modulus of elasticity of a resin-based die is lower compared to that of zirconia crowns (23). Additionally, previous publications have investigated the acoustic response of PMMA. Chen et al. found that PMMA resin has an approximate modulus of elasticity of 2,100.05 ± 114.28 MPa. Furthermore, Chen et al. found that PMMA resin has an approximate modulus of elasticity of 2,100.05 ± 114.28 MPa.

Thus, using PMMA/MZC specimens in load-to-fracture tests can yield clinically relevant outcomes.

Zirconia, a widely used material in dentistry, was chosen as the material for validating SHT due to its technical acoustic properties (21), which makes it suitable for studying crack initiation and propagation in dental ceramics (24) and because of its high flexural strength, enabling effective transmission and propagation of acoustic waves.

Acoustic emission testing is a non-destructive method utilized in various industries, including dentistry, to detect and analyze stress waves resulting from sudden stress redistributions in materials. AET relies on harvesting released energy from the object under examination. Equipped with specialized tools such as sensors, amplifiers, and filters, it collects failing energy data from tested samples (25).

Roques et al. applied AET to analyze bone cement failure through fatigue tests, suggesting AE's usefulness as a preclinical measurement of the strength of cemented implants (26). Silva et al. found that the precision of acoustic testing was comparable with that of micro-CT for detecting cracks in dental ceramics, showcasing its reliability (27). Moreover, Lim et al. studied micro-crack growth in ceramic/dentin interfaces, revealing AET's promise in assessing the integrity of such materials (28). These studies collectively affirm the efficacy of AE methods in detecting material defects, a crucial aspect in ensuring the longevity of dental restorations. However, due to the complexity of dental restorations morphology, adapting ultrasonic receptors to the test setup can be challenging (29).

Our SHT applies AET to dental materials by converting sound-generated vibrations into electrical energy to evaluate material strength and fracture resistance (10, 28).

Because of the complexity of the sample design and size of the crown, the application of ultrasonic receptors to harvest mechanical vibrations was difficult; the vibrating descending indenter was another handicap. The choice was for an audio AET with a sound harvesting system.

By integrating a high-sensitivity microphone within the fracture toughness test, we precisely monitored the noise emissions generated by the samples during loading. The positioning of the microphone at just 1 cm away from the sample ensures optimal sensitivity to detect even subtle crack sounds. This setup, coupled with a custom-designed “cut-off” switch system, enables automatic halting of the load process upon detecting abnormal sounds indicative of crack initiation.

The incorporation of an amplifier and motherboard chipset further augmented the sensitivity and accuracy of the SHT setup (30). By programming the chipset to differentiate between normal machine noise and crack sounds, we ensured reliable detection of crack events during testing. Moreover, the automatic recording of load values in Newton upon the detection of crack sounds enabled efficient data collection and analysis (6, 27). The use of noise-cancellation materials, such as corrugated foam sheets, further minimized external sound interference, thereby controlling environmental variables (31).

While the SHT is generally nondestructive, a minor fraction of the samples catastrophically broke during testing. This could be due to the transmission pathway of the signal from a zirconia crack to a microphone and then through an amplifier to a switch breaker, which involves several variables such as distance, microphone sensitivity, electric wires (XLR,Pig Hog PHM10 8mm®), amplifier quality (93 db), and the speed at which the switch breaker reacts as well material characteristics, including composition, impurities, stress conditions, and specific wave types involved in crack propagation. In addition, sound travels through air at speeds influenced by temperature, humidity, and pressure; at standard conditions, it's about 343 meters per second in dry air, much slower than electrical signals in wires. These electrical signals can nearly reach the speed of light (32).

In the field of dental ceramics, the application of sound harvesting test is relatively unexplored. Our research supports previous findings indicating that the method of crack detection significantly impacts the measured fracture loads in various materials (33, 34). Wang and al.'s work, which shows the superior sensitivity and specificity of acoustic methods over dye penetration for finding cracks in dental ceramics, supports our observations of reduced fracture loads when using sound-based detection (8). Furthermore, Al-Zubaidi and al. and Zhang and al. studies reinforce this perspective, with all reporting that acoustic testing, particularly with the employment of a microphone, is more sensitive than conventional testing (35, 36).

Complementing these acoustic methods, Akono and al. introduced a micro-scratch technique using scratch data for a quantitative assessment of material toughness, offering a highly reproducible and minimally invasive alternative to acoustic and optical assessments (37). Akgün and al. used impulse noise testing to detect defects in ceramic materials, adding another layer to the evolving testing designs (38). These studies suggest that variations in the testing results are influenced by the choice of method, material types, and the specifics of the detection techniques employed, contributing to a broader understanding of how dental materials respond to different testing modalities. Nevertheless, our study adds to the current understanding by examining how brittle dental materials react acoustically.

However, this study is limited to a single type of sample selection. Future studies could include a wider range of brittle materials to enhance the sensitivity of the technique.

## 5 Conclusion

In summary, the sound harvesting methodology outlined in this study presents a promising approach for assessing the fracture resistance of dental ceramics compared to conventional fracture toughness test for identifying early crack formation in brittle materials subjected to stress, thereby allowing for a more accurate values of dental ceramics’ fracture toughness.

The study suggests that variations in the testing results are influenced by the choice of method and the specifics of the detection techniques employed, contributing to a broader understanding of how dental materials respond to different testing modalities.

However, further research on wider range of brittle materials is warranted to support its broader application in dentistry.

## Data Availability

The raw data supporting the conclusions of this article will be made available by the authors, without undue reservation.
